# Respiratory and Neurodevelopmental Outcomes at 3 Years of Age of Neonates Diagnosed with Sleep-Disordered Breathing

**DOI:** 10.3390/jcm13185527

**Published:** 2024-09-18

**Authors:** Bhavesh Mehta, Karen A. Waters, Dominic A. Fitzgerald, Nadia Badawi

**Affiliations:** 1 Department of Neonatology, The Children’s Hospital at Westmead, Sydney, NSW 2145, Australia; nadia.badawi@health.nsw.gov.au; 2 Discipline of Paediatrics & Child Health, Sydney Medical School, University of Sydney, Sydney, NSW 2050, Australia; karen.waters@health.nsw.gov.au (K.A.W.); dominic.fitzgerald@health.nsw.gov.au (D.A.F.); 3 Department of Respiratory Medicine, The Children’s Hospital at Westmead, Sydney, NSW 2145, Australia; 4 Cerebral Palsy Research Institute, Brain and Mind Institute, Sydney, NSW 2050, Australia

**Keywords:** sleep, newborn, sleep-disordered breathing, obstructive sleep apnoea, neurodevelopmental outcomes

## Abstract

**Objectives**: Understanding the long-term consequences of sleep-disordered breathing (SDB) in neonates is crucial. A lack of consensus on diagnostic and treatment thresholds has resulted in limited research in this area. Our study aims to describe the trajectory of SDB in a cohort of high-risk neonates and their respiratory and neurodevelopmental outcomes at 3 years of age, and explore the relationship between SDB during early infancy and neurocognitive outcomes. **Methods**: A retrospectively identified cohort of neonates with moderate–severe SDB were prospectively followed at 3 years of age. Data collected included last polysomnography (PSG) parameters up to the age of 3 years and sleep physician’s recommendations, duration of CPAP use, compliance with treatment, timing of SDB resolution, and neurodevelopmental outcomes. Univariate and multivariate logistic regression analyses were performed to evaluate the association between important respiratory and sleep breathing parameters with the developmental outcomes. **Results**: Eighty neonates were included. Respiratory and developmental outcomes were available for 58 (72.5%) and 56 (70%) patients, respectively. In most patients (47/58, 81%), SDB had resolved by 3 years of age. Survival without major developmental delay was seen in 32/56 (57%), but a significant proportion (21/56, 37.5%) demonstrated global developmental delay. Following univariate analysis, primary diagnosis, apnoea–hypopnoea index (AHI) at the time of last PSG and SDB outcome was significantly associated with developmental delay. However, these associations were not seen in multivariate analysis. **Conclusions**: Despite severity at baseline, SDB resolved in the majority of patients with time and treatment. Although statistically insignificant, logistic regression analysis identified some clinically important associations between neonatal SDB and neurodevelopmental outcomes.

## 1. Introduction

Adequate sleep is essential for a range of biological functions including memory consolidation and cognition [1]. Sleep-disordered breathing (SDB) includes a spectrum of disorders, with obstructive sleep apnoea (OSA) being the most severe form [2]. The estimated prevalence of OSA is 1–4% in children [3,4]. In addition to intermittent hypoxia, sleep disruption/fragmentation with SDB is thought to contribute to poor neurocognitive outcomes [5,6]. In children, SDB has been linked to problems with growth [7], cardiac health [8], and impairment of cognition, attention and executive functions [9,10,11,12,13]. Studies have shown a reversal of some of the deleterious consequences of SDB with treatment in children [14]. However, more information is required regarding equivalent outcomes for neonates and infants.

Previous studies have shown that treatment is not always effective in resolving SDB [15], while a proportion of children will have spontaneous resolution of SDB even without treatment [15,16,17]. Children are also at risk of recurrence of SDB if they develop additional risk factors like adeno-tonsillar hypertrophy or obesity, making it difficult to predict the trajectory of SDB in this population. Although awareness about the risk of SDB and its impact in neonates is increasing [18], its true prevalence remains unknown. The aetiology of SDB in neonates is multifactorial. Maternal OSA during pregnancy can potentially have a negative impact on foetal outcome and neurodevelopment [19]. The management of SDB in neonates is challenging due to the lack of standardised diagnostic criteria, treatment guidelines and normative data. This has led to significant variations in practices worldwide [20,21]. A survey of paediatric sleep medicine physicians [20] from eight different countries revealed a lack of consensus regarding the appropriate cut-off value for defining OSA in neonates and infants with proposed obstructive apnoea–hypopnea index (OAHI) values ranging from 1 to 20 events per hour. Additionally, for infants younger than 2 months, there was no consensus regarding cut-offs used for differentiating mild, moderate, or severe OSA. There was a strong consensus among providers (98%) regarding the need for evidence-based guidelines for infant SDB, underscoring the importance of further research in this area.

There is a lack of information about the natural history of SDB from the neonatal period to early childhood and its impact on the neurodevelopmental outcome. Limited studies have pointed negative links between SDB in neonates and long-term neurodevelopmental outcomes [22,23]. New evidence regarding long-term morbidities associated with residual or untreated SDB in neonates is necessary. The key question is whether the resolution of SDB in infancy improves medium- to long-term neurodevelopmental outcomes.

We hypothesised that the severity of neonatal SDB and/or ongoing residual disease would correlate with worse neurodevelopmental outcomes. The aims of this study were as follows: (1) to demonstrate the trajectory and progression of SDB in a cohort of high-risk neonates through infancy into early childhood, (2) to describe the sleep breathing, respiratory and neurodevelopmental outcomes of this cohort at 3 years of age; and (3) to explore if SDB in neonates is associated with adverse neurodevelopmental outcomes at 3 years of age.

## 2. Materials and Methods

### 2.1. Study Design

In this study, a cohort was retrospectively identified and prospectively followed to 3 years of age.

### 2.2. Setting and Participants

This study consisted of a retrospective review of all neonates <1 month of post-menstrual age (PMA) referred for the management of suspected SDB to a tertiary-level surgical newborn intensive care unit in New South Wales, Australia, between July 2016 and June 2018. Inclusion criteria included neonates >37 weeks post-menstrual age (PMA) and a technically acceptable sleep study. Preterm neonates who were <37 weeks PMA at the time of study were excluded. Eighty neonates were identified from the sleep medicine departmental database and were included in the study. Baseline neonatal data were collected by retrospective chart review, while respiratory and neurodevelopmental outcome data were collected prospectively. A detailed description of the methods, baseline characteristics and hospital outcomes of the study cohort has been previously published [18]. All treatment decisions regarding SDB management were the responsibility of the sleep physician and multidisciplinary team managing the child. All children continued their follow-up as advised by the treating sleep physician. The families of all the children were then approached for a polysomnography (PSG) study and Bayley Scales of Infant and Toddler Development, Third Edition (BSID-III) assessment at 3 years of age. If they could not/did not attend the 3-year assessment, consent was taken to collect outcome data from their medical records. The study protocol was approved by the local ethics committee (LNR/15/SCHN/470).

### 2.3. Respiratory Outcomes

Respiratory outcomes were assessed by PSG, which is the gold-standard test for diagnosing SDB. All patients with respiratory outcome data had a follow-up PSG performed by 3 years of age. For patients who did not attend the 3-year PSG, the result of their last/latest PSG and clinic report was collected from medical records. Data were also collected about the duration of continuous positive airway pressure (CPAP) use and CPAP tolerance data from the sleep technician or sleep physicians’ notes.

All PSGs were attended in hospital and performed using standard channels by a certified sleep technician with standard clinical polysomnography software (Compumedics, Abbotsford, VIC, Australia). All PSGs were reviewed and reported by a paediatric sleep physician using the scoring guidelines of the American Academy of Sleep Medicine (AASM) Manual for the Scoring of Sleep and Associated Events [24,25,26].

SDB severity was classified using the total apnoea–hypopnoea index (AHI). Clinically significant cut-off or a threshold level for treatment in infants and young children to reduce the consequences of SDB remains unclear and contentious. Considering that several patients in our study had their last PSG before 2 years of age, based on suggestions in recent publications [27,28], we used the following classification to try and identify clinically significant SDB: an AHI of <2 was considered normal, an AHI of 2–5 was considered possibly abnormal, an AHI of >5–10 was considered mild, an AHI of >10–15 was considered moderate, and an AHI of >15.0 was considered severe SDB. Other important PSG parameters were also collected and reported.

The status of SDB was recorded based on the sleep physician’s interpretation and recommendation from the last PSG or clinic visit. Respiratory outcome was dichotomised as resolved or unresolved SDB. SDB was considered resolved based on the opinion of the sleep physician, even if it did not match the AHI criteria. The time of resolution of SDB was noted to demonstrate the trajectory of SDB.

### 2.4. Neurodevelopmental Outcome

Developmental outcome was assessed at 3 years of age using the BSID-III. Assessments were completed by two trained assessors who were not blinded to the history but were blinded to current respiratory status. Neurodevelopmental outcome status was collected from medical records if a patient did not participate in the 3-year BSID-III but had an equivalent formal assessment performed by another specialist such as a developmental paediatrician. If there was a new diagnosis of moderate–severe level autism spectrum disorder (ASD), then it was recorded as global developmental delay.

Bayley-III outcomes were defined based on the composite scores with mean of 100 and SD ± 15. Mild delay was defined as a score between 1 and 2 standard deviations below the mean for any domains (cognition, language or motor); moderate delay was defined as between 2 and 3 standard deviations below the mean; and severe delay was defined as >3 standard deviations below the mean. Global delay was used to describe infants who had delay across all areas (motor, cognition, and language) or who had received a diagnosis of moderate–severe level ASD.

### 2.5. Statistical Analysis

Data were analysed using R software for statistical computing (www.R-project.org, accessed on 15 July 2024). Descriptive statistics are presented as a number or percentage, mean with standard deviation (SD) and median with interquartile range (IQR).

Univariate logistic regression analysis was conducted to assess the predictive ability of neonatal clinical parameters and PSG findings on moderate–severe delay (defined as composite score more than 2 SD below the mean) in the three developmental domains or for global delay. A *p*-value < 0.1 in the univariate analysis was deemed significant for inclusion in a stepwise backward multivariate logistic regression analysis to identify the most parsimonious multivariate model. This method systematically removed non-significant variables (*p* > 0.05) based on the Akaike Information Criterion (AIC) until the most statistically robust and parsimonious model was identified. The model’s goodness-of-fit was evaluated using McFadden’s pseudo-R-squared and the Hosmer–Lemeshow tests to assess its adequacy in predicting neurodevelopmental outcomes.

## 3. Results

### 3.1. Study Population

Baseline characteristics of eighty neonates whose data formed the basis for this study are shown in Table 1. The majority (61/80) had associated comorbidities. The study population was divided into three diagnostic groups based on their primary diagnosis or comorbidity—craniofacial group, genetic abnormality group and others. The majority of newborns had severe SDB with a very high mean AHI (62.5 ± 39) and OAI (38.7 ± 34). A significant proportion (56/80, 70%) were discharged home on CPAP. Details of their clinical characteristics, management, and hospital outcome have been published previously [18].

### 3.2. Follow-up Details of Study Cohort

**Figure 1:**
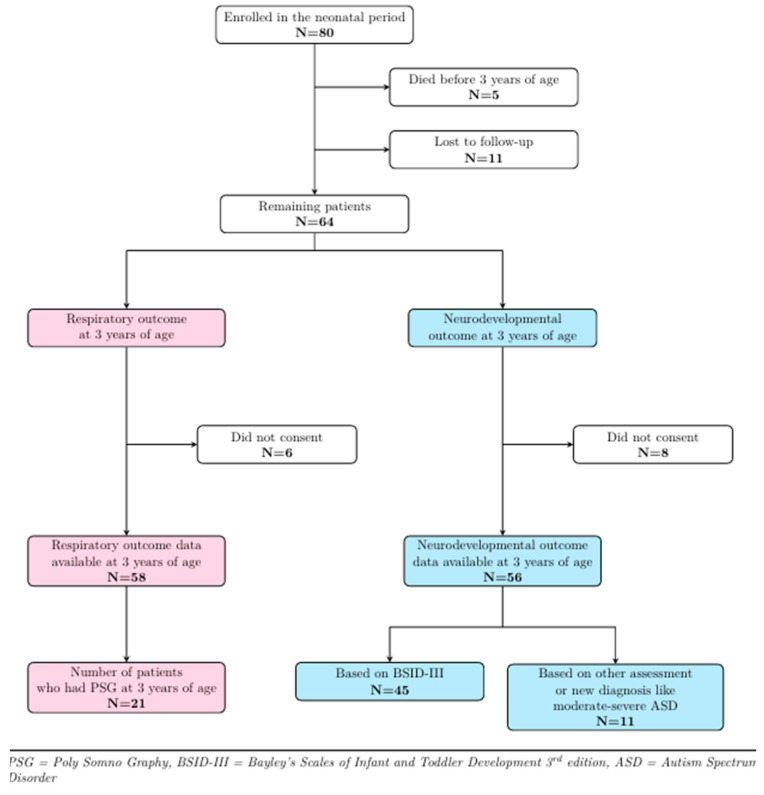
Three-year follow-up details of study cohort.

Figure 1 shows the follow-up details of the study cohort. Of the 80 patients who participated in the baseline study, 5 died before 3 years of age, and 11 could not be contacted, leaving 64 patients eligible for follow-up. Six patients did not consent to respiratory follow-up, while eight declined neurodevelopmental follow-up at 3 years of age, thereby leaving 58 and 56 patients, respectively, with respiratory and developmental outcome data. A total of 21/58 [36%] patients returned for PSG at around 3 years of age, while for the remaining subjects, respiratory outcome data were collected from medical records. Forty-five patients [80%] agreed to participate in the BSID-III assessment, while for 11 patients [20%], outcome data were extracted from medical records. Seven of these patients had their development assessed using the Griffiths Scales of Child Development, while the other four were diagnosed with autism spectrum disorder (ASD).

### 3.3. Respiratory Outcomes at 3 Years of Age

The median age of the last follow-up PSG was 13 (±11.6) months (Table 2), with an age range of 2 to 42 months. A total of 8/58 (14%) children were <6 months old, 24/58 (41%) were between 6 and 24 months old, and 26/58 (45%) were >24 months old at the time of their last PSG. The mean duration of CPAP use was 12.8 (±11.5) months, with an age range of 1 to 42 months. Follow-up data showed that 9/56 (16%) patients did not tolerate CPAP soon after the discharge, while 17/56 (30%) stopped using it at some point before the recommendation from the sleep physician.

SDB status based on AHI criteria at the time of their last PSG was for the majority (40/58, 69% patients) normal or mildly abnormal, while the remaining 18/58 (31%) showed moderate–severe SDB. Sleep physicians’ opinions differed slightly; they interpreted that SDB was resolved in 47/58 (81%) of patients. Out of 11 patients interpreted as having ongoing SDB at 3 years of age, 7 had genetic syndromes, while 2 belonged to each of the craniofacial and other groups. Two patients had an ongoing tracheostomy. Only one patient underwent adenotonsillectomy before the age of three years.

**Figure 2:**
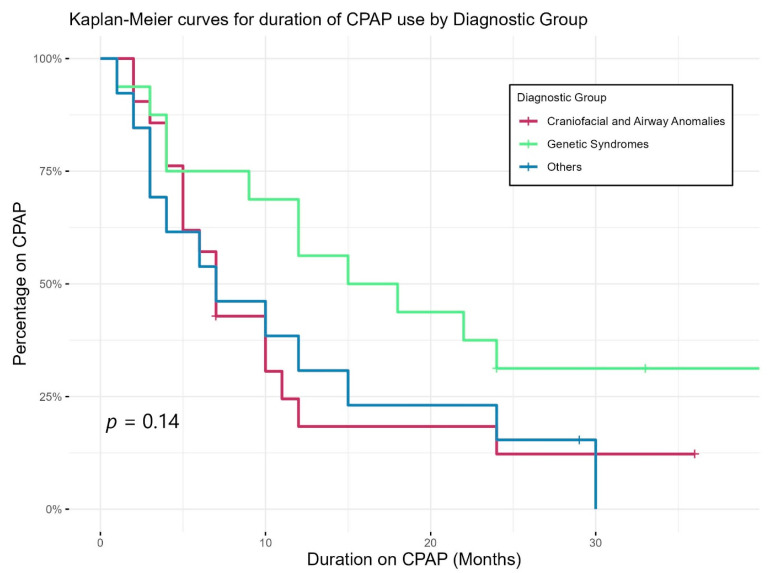
Duration of CPAP use of different diagnostic groups.

Figure 2 shows Kaplan–Meier curves demonstrating the duration of CPAP use of different diagnostic groups. CPAP use lasted longer in those with genetic abnormalities than others; however, the differences in duration did not reach statistical significance. Eight children were still on CPAP by three years of age, two had tracheostomy (one with bilateral vocal cord palsy and one with CHARGE syndrome), while the other six patients had genetic syndromes including skeletal dysplasia and chest wall deformity.

**Figure 3:**
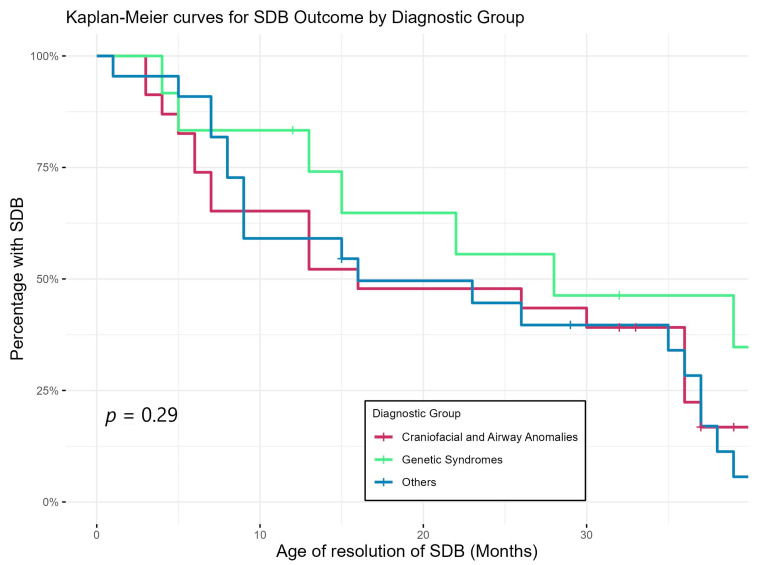
Age of resolution of SDB of different diagnostic groups (based on sleep physicians’ interpretation/report of last PSG).

Figure 3 shows Kaplan–Meier curves demonstrating the trajectory of SDB by showing the timing of resolution of SDB of the three diagnostic groups. This again shows that SDB persisted longer in the group with genetic abnormalities compared to the rest of the cohort but did not reach statistical significance.

Sleep and respiratory parameters from the last PSG are shown in Table 3. Depending on the timing of PSG, the cohort was divided into three groups: last PSG performed before 6 months of age, between 6 and 24 months of age and after 2 years of age. Baseline neonatal PSG parameters are given for comparison. Follow-up data demonstrated expected changes in sleep parameters with increasing age: sleep efficiency increased, percent of REM sleep decreased, and NREM sleep increased over time. Respiratory parameters also showed progressive improvement with increasing age. Respiratory arousal index was normal in all groups [29]. The desaturation index was higher than the reported norms [29] in the <2 years of age groups; however, mean oxygen saturation (SpO2) and percent of total sleep time (TST) with an SpO2 < 90% and mean TcCO2 (transcutaneous carbon dioxide) remained normal in all groups [30]. The percentage of TST spent with TcCO2 > 45 mm Hg was similar to the norms [29]. TcCO2 time >50 mm Hg was higher in the 3-year group, although it was less than 25% of TST, one of the diagnostic criteria for paediatric OSA [4]. AHI, OAI and CAI all decreased significantly over time, although AHI and OAI remained higher than the norms [29,30,31].

### 3.4. Neurodevelopmental Outcomes at 3 Years of Age

The mean age of BSID assessment was 38.0 ± 2.3 months (Table 4), with an age range of 35 to 42 months. The mean composite score for cognition was at the lower end of the normal range, while mean scores for motor and language domains were just below −1 SD. An outcome without any major developmental delay was seen in 32/56 (57%) children. A significant proportion (21/56, 37.5%) had global developmental delays: four of these children were diagnosed as severe-level ASD, while one patient was diagnosed with optic atresia and blindness. A total of 12/21 (57%) children with global delay had a genetic syndrome.

### 3.5. Regression Analyses

The variables analysed in univariate analysis (Table 5) included baseline neonatal sleep and respiratory parameters, gestational age, gender, birth weight, AHI at the time of last follow-up PSG, SDB outcome, duration of CPAP use and diagnostic groups (primary diagnosis). In the univariate analysis, the diagnostic group was significantly associated with both cognitive delay (*p* = 0.04) and global developmental delay (*p* = 0.01). Within the same model, the SDB outcome approached borderline significance for predicting cognitive delay (*p* = 0.06) and motor delay (*p* = 0.05). Additionally, AHI at follow-up PSG showed significant association with language delay (*p* = 0.04). However, in multivariate logistic regression, including all variables with *p* < 0.1 in univariate analyses, none of the variables remained statistically significant at *p* < 0.05 for predicting delay. Moreover, there was no significant difference in neurodevelopmental outcome between neonates with an AHI <10 and AHI >10.

## 4. Discussion

There are significant gaps in the literature regarding SDB in neonates, covering a range of aspects of the disease and including its trajectory with age. Ours is the first study to report the trajectory of SDB and medium-term outcomes in a group of high-risk neonates and explore the relationship between SDB in the neonatal period and developmental outcomes at 3 years of age. Our data showed that despite having severe SDB at baseline, in the majority of patients, it improved and resolved with treatment and time. Our study demonstrates that patient characteristics or phenotypes are important determinants of both respiratory and neurodevelopmental outcomes. Multivariate regression analysis did not reveal any significant associations between SDB and neurodevelopmental outcomes.

The neonates in our study had significant comorbidities, and the majority had severe SDB, requiring a prolonged period of CPAP support (mean duration 12.8 ± 11.5 months). CPAP is reported to be an effective treatment for upper-airway obstruction and is widely used to treat SDB in infants with genetic and muscular problems [32,33,34,35]. A systemic review by Bedi [36] concluded that the long-term use of non-invasive ventilation (NIV) is both effective and feasible in infants with a wide range of underlying problems. Cielo [32] reported that CPAP is well tolerated in infants compared to the school aged children. In our study, nine (16%) patients stopped using it soon after discharge in early infancy. The most common reason given by parents was intolerance of CPAP, including crying or unsettledness in infants. This is consistent with previous studies showing that compliance and a high dropout rate are major issues with CPAP use [32,37,38,39]. Other reasons described for poor CPAP adherence include caregiver factors and mechanical factors, including poor mask fitting and irritation to skin or eyes and nasal congestion [32,37,38]. In 17 (30%) patients, CPAP was well tolerated initially; however, it was electively stopped at some point by the parents before the recommendation of the sleep physician. It is also possible that in these children, improvement in respiratory parameters with time would have led to intolerance of CPAP, as most of these children’s SDB was deemed resolved by the sleep physician in the subsequent follow-up PSG. Our study found no correlation between the length of CPAP use and developmental outcomes (Table 5). Children with genetic syndromes used CPAP for longer durations than those of other groups, consistent with the recent literature [32].

Our study showed a discrepancy between the AHI criteria and physician’s interpretation of the PSG result, with the physicians recommending fewer patients for ongoing/further treatment. This matches with a previous study [40] showing that physicians are likely to incorporate other factors like age, symptoms, other respiratory parameters, treatment tolerance and family/environmental factors when determining the severity and management of SDB in children, especially under 2 years of age. None of the children whose SDB was thought to be resolved/non-significant by physicians returned with SDB symptoms within the study period. Physicians’ global interpretation therefore might be a better predictor of outcomes than strict AHI-based severity alone, considering the holistic view taken by physicians. Based on their interpretation, SDB was resolved in the majority of our patients by the end of the study period. Our resolution rate of 47/58 (81%) is higher than those in previously published studies (20−70%) for older children with mild SDB [41,42]. However, it is similar to the 85% resolution rate reported in a study of infants with OSA [43]. Resolution took longer in the genetic group compared to others (Figure 3), consistent with previous studies showing that genetic conditions, such as Trisomy 21, are usually associated with multi-level anatomic upper-airway obstruction with a higher rate of persistent OSA after treatment [44,45]. Our study results suggest a potential relationship between SDB outcomes and motor and cognitive delays (Table 5). Biggs and colleagues [46] found that preschool children originally diagnosed with SDB, regardless of treatment outcome, had worse cognitive and behavioural functioning than controls.

We chose conservative modified AHI criteria to classify SDB compared to what is commonly used in children [47,48]. A significant proportion of our study cohort had their last PSG performed before 2 years of age. Applying the paediatric classification of SDB to children < 2 years of age would result in a higher severity rating of SDB [40]. Another issue is uncertainty about the benefit of treatment of children who have an AHI ≥1 but <5 events per hour of sleep. Previous studies have shown no difference in SDB sequelae between a child that has an AHI < 1 event/h and one who has an AHI between 1 and 5 events/h [49,50]. A recent survey of sleep physicians indicated a lack of agreement amongst them regarding the threshold criteria for treatment [20]. Brooks [28] suggested modifying the current SDB severity classification to make it more useful for treatment outcome with more focus on physiology and outcome. This was also supported by the study from DeHaan [40]. We wanted to clearly identify children with clinically significant SDB who had a therapeutic need. From the literature, an AHI value of at least 5 is considered more suggestive of this need [28]. We therefore decided to use AHI >5 as a cut-off for an abnormal PSG.

We divided the cohort in three groups for the evaluation of follow-up PSG parameters. Infants < 6 months were a distinct subgroup with respect to diagnosis and treatment recommendations, and we wanted to ensure that our analysis reflected the age-related changes in sleep and respiration. This was demonstrated by PSG findings, as sleep parameters differed by age group as expected (Table 3). Younger (<6 months) patients had an increased arousal index as well as slightly poorer sleep efficiency (75–81%) compared to normal children (80% or more) [29,30,31]. However, these parameters normalised with increasing age. Normative data in infants are important in deciding whether this elevated arousal index is a function of age or a manifestation of SDB. The respiratory arousal and desaturation indices also showed progressive improvement with age. Although AHI, OAI and CAI showed significant improvement with increasing age, they remained higher than the norms [4,27,30]. This may either represent some ongoing residual SDB or show ongoing resolution with time, as baseline AHI was very high to begin with. This study reinforces the view that an experienced sleep physician considers factors outside respiratory parameters when making management decisions. While respiratory events are the primary PSG criteria used to define SDB, further work is needed to determine whether evidence of sleep disruption influences physicians’ assessment and management of SDB in younger children.

A significant proportion (24/56, 43%) of the study cohort showed major developmental delay at 3 years of age. Patient-specific factors played a key role in neurodevelopmental outcomes. Although in univariate analysis, we found significant effects of some factors on development, they failed to reach statistical significance in multivariate analysis. It is possible that our small sample size may have limited our ability to detect this association; however, this is no different from limited studies that have also reported negative links between SDB in neonates and neurodevelopmental outcomes [22,23]. However, this is different from the study by Piteo [51], which reported that snoring in the first 6 months of life was associated with lower cognitive scores. Bigg [46] also reported a significant effect of SDB on several neurocognitive and behavioural domains in preschool children. Smith and colleagues [52] reported that infants with cleft lip/palate with more obstructive episodes had lower global behaviour scores at 3 years of age.

### Limitations of This Study

We acknowledge that the relatively small sample size reduced the statistical power of our regression model, potentially limiting its ability to accurately predict outcomes. Therefore, these results should be interpreted with caution. Our study comprises a cohort of high-risk infants who had confounding factors/diagnoses that are known predictors of poor neurodevelopmental outcome. Approximately a quarter of patients (21/80, 26%) had genetic syndromes, potentially accounting for the high rate of significant developmental delays in our cohort. The potential for bias towards worse outcomes exists due to the higher rate of associated comorbidities in our cohort.

The data collected were from a single centre, with the cohort representing the severe spectrum of SDB, suggesting a referral bias. Follow-up was completed for about 70% of the original cohort, raising the possibility of selection bias. It is possible that parents who refused to undergo repeat PSG at 3 years might have been under the impression that their child’s SDB was resolved. For these patients, parents’ view and lack of re-presentation to our sleep department or to another specialist by 3 years of age was taken as a proxy for the resolution of significant SDB. Conversely, families who had concerns about their children may have been motivated to participate in a follow-up. We may have missed surgical intervention in some patients, which might have led to SDB resolution. Repeat PSG data were not available at 3 years of age for all patients prior to BSID-III testing to confirm the persistence or resolution of SDB at that time point. We did not have serial polysomnograms to permit trajectory analyses of sleep data over time for individual patients.

Despite these limitations, our study contributes to a small but growing body of literature aimed at improving our understanding of the history and impact of SBD in neonates leading into the preschool years. This study has several strengths. It demonstrates the trajectory and outcome of SDB in a diverse group of neonates with various conditions, while limited prior studies have focused on single groups [22,23,52]. Additionally, all patients with respiratory data underwent follow-up polysomnography (PSG), the gold standard for SDB diagnosis. Moreover, this study cohort was larger than in comparable studies [22,23,52] and represents the longest follow-up of neonates with SDB to date.

## 5. Conclusions

Our study suggests that clinically important associations may be present between SDB in the neonatal period and later developmental outcomes (at 3 years of age). Establishing definitive associations may require a larger sample. Additional research is necessary to understand the relationship between SDB severity, treatment effectiveness, and outcomes in non-syndromic infants. Furthermore, the inconsistency in defining thresholds for SDB in neonates, infants and young children hinders the ability of studies to effectively compare the presence and absence of SDB with outcomes of interest. A coordinated effort to define clinically significant SDB thresholds in this at-risk patient population is required.

## Figures and Tables

**Table 1:** Baseline demographic, clinical and respiratory parameters of study cohort in the neonatal period.

Study Cohort (*n* = 80)	Value (N%)
**Demographic details**	
Gestational age (weeks) (Mean ± SD)	36.7 ± 4.1
Birth weight (grams) (Mean ± SD)	2793.5 ± 882.8
Preterm–N (%)	24 (30%)
Males–N (%)	54 (67.5%)
**Diagnostic group based on primary comorbidity**	
Craniofacial and airway anomalies group	30 (37.5%)
Genetic syndromes group	21 (26.5%)
Others	29 (36%)
Chronic lung disease	6
Neurological	3
Cardiac	1
No obvious risk factors	19
**Abnormal chromosome microarray**	14 (17.5%)
**Abnormal cerebral imaging**	17 (21.5%)
**Baseline SDB parameters of neonates**	
Number diagnosed with SDB	73 (91%)
AHI of cohort (Mean ± SD)	62.5(39)
OAI of cohort (Mean ± SD)	38.7(34)
**Hospital outcome**	
Died in hospital	1
Underwent airway surgery	8 (10%)
Discharged home on CPAP	56 (70%)
Discharged home on oxygen	2
Discharged home on apnoea monitor	11 (13.8%)

SDB—sleep-disordered breathing, PSG—polysomnography, CPAP—continuous positive airway pressure, AHI—apnoea–hypopnoea index, OAI—obstructive apnoea index, SD—standard deviation.

**Table 2:** Respiratory outcome data at 3 years of age.

**Age at the time of last follow-up (months) (*n* = 58)**	
Mean ± SD	13.02 ± 11.6
Median (IQR)	7 (4–18)
**Duration of CPAP use (months)**	
Mean ± SD	12.8 ± 11.5
Median (IQR)	7 (4–18.5)
**CPAP use/tolerance (*n* = 56)**	
Did not tolerate CPAP and stopped soon after the discharge (<3 months of discharge)	9 (16%)
Tolerated CPAP well for several months but stopped before the next scheduled follow-up PSG/before recommendation from sleep physician	17 (30%)
Tolerated CPAP well and stopped after the normal follow-up PSG and sleep physician’s recommendation	25 (45%)
Died before 3 years	5 (9%)
**Status of SDB according to AHI criteria based on the last PSG (*n* = 58)**	
Normal	9 (15.5%)
Possibly normal	16 (27.5%)
Mild SDB	15 (26%)
Moderate SDB	9 (15.5%)
Severe SDB	9 (15.5%)
**Status of SDB according to sleep physician’s interpretation based on the last PSG (*n* = 58)**	
SDB Resolved	47 (81%)
SDB Unresolved	11 (19%)

CPAP—continuous positive airway pressure, PSG—polysomnography, SDB—sleep-disordered breathing, SD—standard deviation, IQR—interquartile range, AHI—apnoea–hypopnea index.

**Table 3:** Follow-up sleep and respiratory parameters—data based on the last PSG performed by 3 years of age vs. baseline PSG in the neonatal period.

	Baseline PSG	Last Follow-up PSG		
PSG Parameters	Neonatal Data * n = 67	At Less Than 6 Months of Age *n* = 8	At 6–24 Months of Age *n* = 24	At 2–3 Years of Age *n* = 26
TST (minutes)	249.3 ± 55.2	418.9 ± 85.3	420.4 ± 113.8	409.1 ± 103.1
Sleep efficiency (%)	68.9 ± 12.0	75.1 ± 15.8	81.0 ± 10.4	80.6 ± 10.6
REM (% of TST)	54.5 ± 13.2	38.8 ± 11.1	34.3 ± 10.7	22.6 ± 7.2
Stage 2 NREM (% of TST)	38.3 ± 13.2	50.2 ± 10.5	50.6 ± 17.3	54.2 ± 20.3
Arousal Index (n per hour)	28.9 ± 8.4	26.0 ± 9.7	12.3 ± 7.2	13.1 ± 5.5
Respiratory Arousal Index (n per hour)	6.3 ± 4.3	3.1 ± 2.4	1.4 ± 1.2	1.6 ± 1.7
Desaturation Index (n per hour)	38.1 ± 32.1	15.0 ± 12.1	11.8 ± 6.5	3.7 ± 3.4
Mean SpO2%	97.1 ± 3.3	98.0 ± 1.0	96.8 ± 1.5	96.5 ± 1.3
SpO2 < 90 (%TST)	3.7 ± 9.6	0.2 ± 0.3	0.1 ± 0.3	0.1 ± 0.3
Mean TcCO2	46.2 ± 7.1	40.8 ± 4.1	43.2 ± 4.7	45.2 ± 5.8
TcCO2 > 45 mm Hg (% TST)	22.0 ± 25.6	3.3 ± 7.3	20.2 ± 24.7	24.1 ± 26.8
TcCO2 > 50 mm Hg (%TST)	24.8 ± 32.4	0.0 ± 0.0	1.7 ± 3.5	20.4 ± 32.3
AHI (n per hour)	58.1 ± 39.6	19.6 ± 18.6	11.5 ± 11.7	4.8 ± 4.3
OAI (n per hour)	38.3 ± 34.3	5.6 ± 5.5	5.1 ± 8.6	3.1 ± 3.7
CAI (n per hour)	9.1 ± 10.9	10.4 ± 12.3	4.0 ± 2.8	1.3 ± 1.2
Central Apnea (seconds)	5.5 ± 2.0	6.4 ± 0.8	7.2 ± 1.7	9.4 ± 4.3
Hypopnea Index	8.1 ± 7.1	3.5 ± 2.5	1.7 ± 1.5	1.6 ± 4.0

PSG—polysomnography, TST—total sleep time, REM—rapid eye movement, NREM—non-rapid eye movement, TcCO_2_—transcutaneous carbon dioxide, AHI—apnoea–hypopnea index, SpO2—saturation of oxygen, OAI—obstructive apnoea index, CAI—central apnoea index, Values represent means ± SD. * Neonatal studies were daytime studies, whereas subsequent studies were overnight studies.

**Table 4:** Neurodevelopmental outcome data at 3 years of age.

**Bayley Scales of Infant and Toddler Development (3rd Edition) (*n* = 45)**	
Age of assessment (months)	
Mean ± SD	38.0 ± 2.3
Median (IQR)	37.0 (36–40.5)
**Bayley Scores Summary**	
Cognitive composite score (Mean ± SD)	87.8 ± 17.0
Language composite score (Mean ± SD)	84.3 ± 19.6
Motor composite score (Mean ± SD)	82.9 ± 20.9
**Neurodevelopmental outcome status at three years of age (*n* = 56):**	
Normal	28 (50%)
Mild delay	4 (7.5%)
Moderate–severe delay	3 (5%)
Global DD	21 (37.5%)
**Survival without major disability (*n* = 32)**	

DD = developmental delay, SD = standard deviation, IQR = interquartile range.

**Table 5:** Factors associated with developmental delay in univariate logistic regression analysis *p*-values.

Variable	Cognitive Delay	Language Delay	Motor Delay	Global DD
Gestation	0.85	0.28	0.56	0.66
Birth Weight	0.88	0.23	0.59	0.94
Gender	0.14	0.47	0.09 *	0.83
AHI	0.55	0.35	0.3	0.17
OAI	0.6	0.47	0.34	0.16
Central AI	0.98	0.32	0.91	0.7
RAI	0.33	0.59	0.5	0.53
T_c_CO2 > 45 mm Hg (%TST)	0.19	0.43	0.34	0.38
SpO2 < 90 (%TST)	0.18	0.46	0.31	0.11
Sleep efficiency (%)	0.55	0.3	0.57	0.54
Follow-up AHI (Last PSG) #	0.22	0.04 *	0.4	0.37
SDB Outcome	0.06 *	0.28	0.05 *	0.12
Duration of CPAP use	0.71	0.68	0.97	0.18
Diagnostic group	0.04 *	0.28	0.99	0.01 *

Note: * indicates *p* < 0.1, # except follow-up AHI, all other sleep and respiratory data are from the baseline cohort. DD—developmental delay, SDB—sleep-disordered breathing, AHI—apnoea–hypopnea index, OAI—obstructive apnoea index, RAI—respiratory arousal index, T_c_CO2—transcutaneous carbon dioxide, TST—total sleep time, PSG—polysomnography, CPAP—continuous positive airway pressure.

## Data Availability

The data presented in this study are available on reasonable request from the corresponding author due to privacy concerns.
